# Antimicrobial Susceptibility Patterns of *Pseudomonas aeruginosa* from Diabetes Patients with Foot Ulcers

**DOI:** 10.1155/2011/605195

**Published:** 2011-11-17

**Authors:** Tamil Selvi Sivanmaliappan, Murugan Sevanan

**Affiliations:** ^1^Department of Microbiology, Dr. N.G.P. Arts and Science College, Coimbatore, Tamil Nadu 641048, India; ^2^Department of Biotechnology, School of Biotechnology and Health Sciences, Karunya University, Karunya Nagar, Coimbatore, Tamil Nadu 641 114, India

## Abstract

*Pseudomonas aeruginosa* is an invasive organism that frequently causes severe tissue damage in diabetic foot ulcers. A major problem in *P. aeruginosa* infection may be that this pathogen exhibits a high degree of resistance to a broad spectrum of antibiotics. The study aimed to isolate and determine the antimicrobial susceptibility patterns of the *P. aeruginosa* population from diabetes patients with foot ulcers attending tertiary care hospitals in and around Coimbatore and their antimicrobial susceptibility pattern. The study was carried out at the Department of Microbiology, Dr. N.G.P. Arts and Science College, Coimbatore, for a period of one year (June 2006 to April 2007). The present study comprised 270 pus specimens collected from diabetic patients with foot ulcers. All pus samples were subjected to gram staining; bacterial culture and subsequently the antibiotic sensitivity to 15 different antibiotics for the confirmed *P. aeruginosa* were performed as per the standard procedures. Eighteen strains (14.28%) of *P. aeruginosa* from 270 diabetic foot ulcers were detected. Almost all the strains exhibited a varying degree of resistance to the antibiotics tested. Multidrug resistance for about 8 to 11 antibiotics was observed among the 55.5% of the isolates. Disk diffusion results show 100% resistance to ampicillin, cefoperazone, erythromycin, norfloxacin, and only cefotaxime, ciprofloxacin exhibited greater activity against *Pseudomonas aeruginosa*.

## 1. Introduction

Diabetes is a chronic disorder that affects a large segment of population and is a major public health problem. Diabetes and foot problems are almost synonymous [1]. A recent WHO report indicates that India has the largest diabetic population (19 million in 1995) that is expected to rise to 57 million by 2025 [2]. A commonly accepted definition of foot infection is the presence of systemic signs of infection (e.g., fever and leucocytosis) or purulent secretions or two or more local symptoms or signs (redness, warmth, indurations, pain, or tenderness) [3]. Viswanathan et al. [4] reported that 25% of diabetic individuals are anticipated to develop severe foot problems at some point in their lifetime that often end with amputation. Diabetic foot infections are more severe and more difficult to treat than infections in nondiabetics. Polymicrobial etiology has been implicated in the infected diabetic foot. Gram-negative infections are three-times more frequent in the diabetic than in non-diabetic individuals [5]. 

Most infections with *Pseudomonas* species occur in compromised hosts. The pathogenicity of these organisms is based on its ability to produce a variety of toxins and proteases and also on its ability to resist phagocytosis [6]. *Pseudomonas aeruginosa* is commonly resistant to antibiotics, and because of this it is a dangerous and dreaded pathogen. The only antibiotic agents to which strains are regularly sensitive are cephalosporins, carbenicillin, colistin, gentamicin, polymyxin, quinolones, and streptomycin; however degrees of cross-resistance between these agents have been reported [7]. *P. aeruginosa* is one of the most important microorganisms that cause clinical problems resulting from high-resistance to antimicrobial agents. Though it is rarely found in the normal flora of humans, it is frequently isolated from patients with burns, cystic fibrosis, and neutropenia [8].* P. aeruginosa *may cause severe tissue damage in diabetics and should never be ignored as insignificant in diabetic foot ulcers. Moreover, it should never be considered a contaminants or normal flora, and it should clearly be considered a pathogen, because it may result in sepsis and amputation [9]. 

One of the challenges in managing *P. aeruginosa* infections is an inherent resistance mechanism, referred to as intrinsic resistance. Its multiplicity of resistance mechanisms may render this microbe less amenable to control by antibiotic cycling [10]. *P*. *aeruginosa* is noted for its metabolic versatility and its exceptional ability to colonize a wide variety of environments and also for its intrinsic resistance to a wide variety of antimicrobial agents. The bacillus almost never causes infections in healthy individuals and often infects the immunocompromised. Because of its virulence and the limited choices of effective antimicrobial agents, treatments of infections by *P. aeruginosa* are often difficult [11]. Though a lot of work has been carried out elsewhere pertaining to *P. aeruginosa*, the prevalence and the antimicrobial susceptibility patterns of *P. aeruginosa* from diabetes patients with foot ulcers have rarely been documented in this part of South India. Therefore, the present study has been carried out to study the prevalence of *P. aeruginosa* and their antimicrobial susceptibility by the Kirby Bauer-Disk Diffusion method.

## 2. Materials and Methods

### 2.1. Sample

The study was based on 270 pus specimens received for the screening of *P. aeruginosa* from diabetes patients with foot ulcers attending tertiary care hospitals in and around Coimbatore. Specimens included in the study were from “Soft tissue infection” which includes foot wound and “limb threatening infections” specimens were included in the study. Specimens were obtained using aseptic techniques to avoid contamination and were promptly transported to the laboratory in a sterile swab in ice-cold conditions.

### 2.2. Isolation and Identification of Pseudomonas aeruginosa

The isolation and identification of test organisms was carried out by the procedures suggested by Valentina and Lalitha [12]. Identification analysis like Gram staining, motility, catalase, oxidase, pigment production, growth on cetrimide agar, ability to grow at 42°C, gelatin hydrolysis, arginine dihydrolase, acid from Hugh-Leifson's glucose, and nitrate reduction tests were carried out.

### 2.3. Antibiotic Sensitivity Testing (Kirby Bauer-Disk Diffusion Method)

Antibiogram was performed using commercially available antibiotic discs (Hi-Media, Mumbai) with a standard *P. aeruginosa* ATCC 27853 as a positive control. Kirby-Bauer, recommended by the CLSI [13], was used for antimicrobial susceptibility testing. The identified 18 *P. aeruginosa* strains were tested against ampicillin (10 *μ*g), amikacin (30 *μ*g), ceftazidime (30 *μ*g), cefotaxime (30 *μ*g), ciprofloxacin (5 *μ*g), cefoperozone (75 *μ*g), co-trimoxazole (25 *μ*g), erythromycin (10 *μ*g), gentamicin (10 *μ*g), imipenem (10 *μ*g), norfloxacin (10 *μ*g), piperacillin (100 *μ*g), tobramycin (30 *μ*g), ticarcillin (75 *μ*g), and tetracycline (30 *μ*g).

## 3. Results and Discussion

### 3.1. Patients

Of the total 270 diabetic patients suffering from foot infections, 180 were male and 90 were female. The male-to-female ratio was 2 : 1 and the age of the patients ranged between 36 to 75 years. Very few of these patients have undergone amputation. Studies conducted in Chennai have shown that males were more susceptible than females in the ratio of 8 : 3 [14]. Previous studies have shown that males were more susceptible than females in the ratio of 2 : 1, which is in accordance with the current study. Predominance of male over female patients as shown in the study can be explained by the fact that in our country males are exposed more to the outside environment because of their mobility as compared to females.

### 3.2. Isolation Rate

Of the 270 pus specimens of diabetic patients with foot infections, 180 (66.6%) specimens were culture positive and the other 90 (33.3%) were negative. Among the strains, aerobic gram-negative *Pseudomonas *species were 126 (70.0%) and other aerobic organisms comprised 54 (30.0%). From the 126* Pseudomonas *species, 18 (14.30%) were found to be *P. aeruginosa.* Dhanasekaran et al. [5] reported the prevalence of *Pseudomonas* species to be 18.79% from a diabetic centre in Chennai. In a similar study conducted in a private hospital in Chennai, 29.8% strains among diabetic foot ulcer patients were *P. aeruginosa *[15]. This finding shows the high prevalence of* Pseudomonas* species and *P. aeruginosa* among diabetes patients with foot ulcers.

### 3.3. Antibiogram Pattern of Pseudomonas aeruginosa

**Figure 1:**
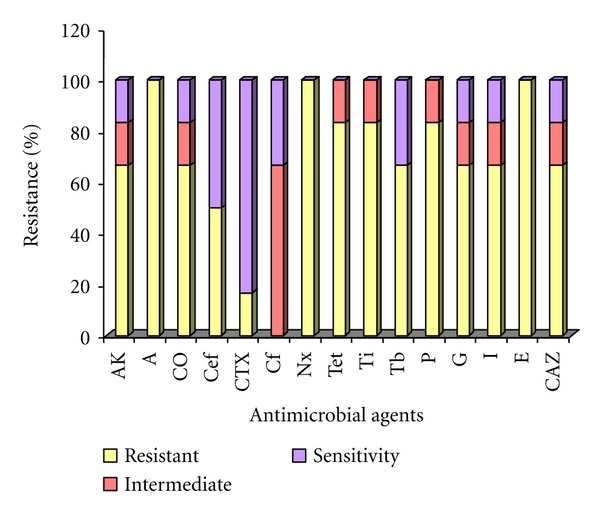
Antimicrobial susceptibility patterns of *Pseudomonas aeruginosa* among diabetes patients with foot ulcers.

The Mueller Hinton agar-based antibiogram-resistogram pattern study of *P. aeruginosa* isolated from foot ulcers of diabetes patients is shown in Figure 1. Almost all of the eighteen *P. aeruginosa *strains screened showed 100% resistance to ampicillin, erythromycin, and norfloxacin, similarly 83.3% resistance to piperacillin, ticarcillin, and tetracycline, 66.6% resistance to ceftazidime, imipenem, gentamicin, amikacin, tobramycin, and cotrimoxazole and 50.0% resistance to cefoperazone. However, 15 (83.3%)* P. aeruginosa *strains were susceptible to cefotaxime. Multidrug resistance for about 8 to 11 antibiotics was observed among 55.5% of the strains (Table 1). No single antibiotic showed 100% sensitivity to all *P. aeruginosa* strains. Resistance was least with cefotaxime (16.6%), followed by an intermediate resistance of 66.7% observed for ciprofloxacin.

India has the largest number of diabetic individuals and appreciably poor economic conditions; the study on this intrinsic resistant organism in diabetic foot infections assumes significance. The present study has shown the incidence of *P. aeruginosa* to be 14.3% in diabetic foot ulcers, which is significant when compared to previous studies. In accordance with earlier observations [16], the current study has demonstrated that *P. aeruginosa *strains isolated from foot ulcers are more resistant to antimicrobial agents. This may be due to the fact that the strains isolated from clinical specimens have been subjected to the selective actions of both disinfectants and antibiotics [16]. As expected, the strains were resistant to imipenem, piperacillin, erythromycin, ticarcillin, tetracycline, gentamicin, co-trimoxazole, and amikacin indicating the emergence of multidrug-resistant strains. Antibiogram also revealed that cefotaxime and ciprofloxacin retained high levels of antipseudomonal activity and cefoperazone, gentamicin, ceftazidime, amikacin, imipenem, and tobramycin had the least activity. Ciprofloxacin and cefotaxime were found to be better choices for diabetes patients with foot ulcers in this part of the region when compared to gentamicin, imipenem, piperacillin, and other third-generation cephalosporins. The present study thus revealed the importance of *P. aeruginosa* from diabetes patients with foot ulcers, which is necessary for proper management of diabetes patients.

## Figures and Tables

**Table 1:** Multidrug resistance patterns of *P. aeruginosa* of diabetic foot ulcers.

No. of drugs resistant	No. of isolates (*n* = 18)	Resistance (%)
≥8	18	100
≥9	15	83.3
≥10	13	72.2
≥11	10	55.5
≥12	7	38.8
≥13	5	27.7
≥14	2	11.1
≥15	0	0
